# Sodium Alginate-Based Antibacterial Coatings Reinforced with Quaternized Lignin–Cinnamaldehyde Composite Particles for Fruit Preservation

**DOI:** 10.3390/foods14244203

**Published:** 2025-12-07

**Authors:** Jianshuo Miao, Yuanrong Lai, Yidan Zhang, Jiapeng Wei, Kehao Fan, Ningjing Sun, Zhiyong Qin

**Affiliations:** 1 College of Resources and Environment Sciences, Baoshan University, Baoshan 678000, China; mjs1790@163.com; 2 School of Resources, Environment and Materials, Guangxi University, Nanning 530004, China; yuanronglai@163.com (Y.L.); zhangyidanegg@163.com (Y.Z.); plianasp@163.com (J.W.); fankehaoo@163.com (K.F.)

**Keywords:** quaternary ammonium lignin–cinnamaldehyde composite particles, sodium alginate, fruit preservation, biodegradable coating, antimicrobial activity

## Abstract

Sodium alginate (SA) is widely used as an edible coating for fruit preservation, but its weak water barrier and antibacterial properties limit broader application. In this study, quaternary ammonium lignin–cinnamaldehyde (QKC) composite particles were incorporated into SA as multifunctional fillers to construct antibacterial coatings. Electrostatic and hydrogen-bonding interactions between cationic QKC and anionic SA yielded a uniform, stable network with improved hydrophobicity and UV-shielding capacity. At 5 wt% QKC loading (SA5), the tensile strength increased from 11.53 to 24.42 MPa (111.8% higher than SA0), while water vapor permeability decreased by 35.4%. SA coatings also exhibited strong antioxidant activity, and the ABTS radical scavenging rate increased to 70.22% at 7 wt% QKC, with SA5 offering a favorable balance between antioxidant, barrier, and mechanical properties. SA5 showed pronounced antibacterial efficacy, giving inhibition rates of 96% against *Staphylococcus aureus* and 65% against *Escherichia coli.* Coating trials on persimmons and tangerines demonstrated that SA5 reduced weight loss, delayed firmness decline, and mitigated decay during storage. In addition, calcium-crosslinked SA/QKC hydrogel beads markedly delayed visible mold growth on blueberries. These results indicate that QKC-reinforced SA coatings provide a promising strategy for enhancing the postharvest quality and shelf life of fresh fruit.

## 1. Introduction

Preservative materials are crucial for fruit preservation, yet conventional petroleum-based plastics pose significant challenges as they are poorly degradable, contributing to environmental pollution [1], and pose potential human health risks [2,3]. Furthermore, the thermal or photodegradation of these synthetic polymers often leads to the emission of toxic byproducts, including phthalates and bisphenol A [4,5]. These issues have intensified the urgency to develop biodegradable alternatives for food preservation. Compared with petroleum-based plastics, biodegradable polymers offer environmental compatibility, shorter degradation cycles, and improved safety profiles [6,7]. Recent advances in green chemistry and polymer modification have expanded the use of chitosan [8], polylactic acid [9], proteins [10], and cellulose [11] as packaging or coating matrices, which are abundant, non-toxic, and effective in preservation applications, making them leading alternatives to petroleum-based materials.

Among these candidates, sodium alginate (SA), a natural anionic polysaccharide extracted from brown algae, is particularly promising due to its excellent biocompatibility [12], film-forming properties [13], and inherent biodegradability [14]. Its backbone comprises β-D-mannuronic acid (M) and α-L-guluronic acid (G) residues linked by 1,4-glycosidic bonds; abundant hydroxyl and carboxyl groups render SA highly hydrophilic. As a neat film, SA readily absorbs water, swells, and peels, and its water vapor barrier capacity is low [15], limiting its ability to reduce moisture loss [16]. In addition, SA lacks strong antioxidant moieties and cationic or amine functionalities, resulting in weak antioxidant and antibacterial performance [17]. To address these limitations, several strategies have been developed, including blending with other biopolymers [18], chemical or ionic crosslinking [19], incorporating functional active compounds, and constructing multilayer coatings. Among these, the integration of antimicrobial or antioxidant components has been widely adopted [20]. Therefore, to overcome these limitations, an ideal modifier for SA coatings is urgently needed. Such a modifier must simultaneously possess hydrophobicity (to enhance the water vapor barrier), reinforcing properties (to improve mechanical strength), and potent, broad-spectrum antimicrobial activity.

Among the many bio-based candidates, lignin and cinnamaldehyde (CIN) have garnered significant attention, but they present distinct challenges when used individually. Lignin, an abundant byproduct of the paper industry, can be chemically modified (e.g., quaternization, QL) to impart antimicrobial properties and enhance compatibility [21,22]. However, its efficacy as a standalone antimicrobial agent in coatings can be limited. On the other hand, CIN is a natural essential oil component recognized for its potent, broad-spectrum antimicrobial activity against various foodborne pathogens [23,24]. The primary obstacle hindering its application is its high volatility and instability, which leads to a rapid loss of function and can impart a strong odor, compromising the coating’s durability and sensory acceptability.

A promising strategy to overcome these individual drawbacks is to fabricate composite particles (QKC) by loading CIN onto QL. In this design, the quaternized lignin (QL) is hypothesized to act as a novel carrier or anchor for cinnamaldehyde (CIN). This combination is expected to create synergistic effects: (1) QL can effectively encapsulate CIN, reducing its volatility and enabling a controlled, sustained release of the antimicrobial agent; (2) The QL carrier itself possesses antimicrobial properties, which, combined with CIN, may broaden the antimicrobial spectrum or enhance its potency; (3) The resulting QKC composite particles, acting as a multifunctional filler, are expected to improve the mechanical and hydrophobic barrier properties of the SA matrix through enhanced interfacial interactions (e.g., hydrogen bonding and electrostatic interactions).

To the best of our knowledge, while the individual applications of lignin and cinnamaldehyde in food preservation have been explored, the development of quaternized lignin-cinnamaldehyde (QKC) composite particles as a novel, multifunctional filler to simultaneously enhance the mechanical, water barrier, and synergistic antimicrobial properties of sodium alginate edible coatings has not yet been reported.

Therefore, this study aimed to design and fabricate a novel SA-based composite coating enhanced with quaternized lignin-cinnamaldehyde (QKC) particles. The structural characteristics, physicochemical properties (e.g., mechanical, water barrier, and thermal stability), and synergistic antimicrobial activities of the composite coatings were systematically investigated. Furthermore, the practical application potential of the optimized coating (SA5) for preserving postharvest persimmons, tangerines, and blueberries was evaluated by monitoring key quality parameters during storage.

## 2. Materials and Methods

### 2.1. Materials

Kraft lignin (KL) was purchased from Shandong Sun Paper Industry Joint Stock Co., Ltd. (Jining, Shandong, China). Sodium alginate (SA, viscosity: 200–550 mPa·s at 1% solution, 20 °C), 2,3-epoxypropyltrimethylammonium chloride, 2,2-diphenyl-1-picrylhydrazyl, 2,2′-azino-bis(3-ethylbenzothiazoline-6-sulfonic acid) diammonium salt, cinnamaldehyde, glycerol, and sodium hydroxide (NaOH) were purchased from Macklin Co., Ltd. (Shanghai, China). *Staphylococcus aureus* (*S. aureus*) and *Escherichia coli* (*E. coli*) were purchased from Guangdong Huankai Biochemical Technology Co., Ltd. (Shaoguan, Guangdong, China). All chemicals were of analytical grade and were used without further purification. Deionized water was used in all experiments.

### 2.2. Preparation Method

#### 2.2.1. Preparation of Quaternary Ammonium Lignin-Cinnamaldehyde Particles

QKC composite particles were fabricated based on protocols established in our prior work [13], with minor modifications. Briefly, a reaction mixture was prepared by dissolving Kraft lignin (KL, 10 g) and 2,3-epoxypropyltrimethylammonium chloride (GTAC, 10 g) into a NaOH solution (50 mL, 0.6 M). This mixture was agitated at 75 °C for 1 h. The resulting product was purified via dialysis (MWCO: 3500 Da) and subsequently lyophilized to yield quaternized kraft lignin (QKL). To prepare the composites, 3 g of the obtained QKL was dissolved in deionized water (100 mL), followed by the addition of cinnamaldehyde (3.75 g).

#### 2.2.2. Preparation of Sodium Alginate Coating Containing QKC Particles

**Figure 1:**
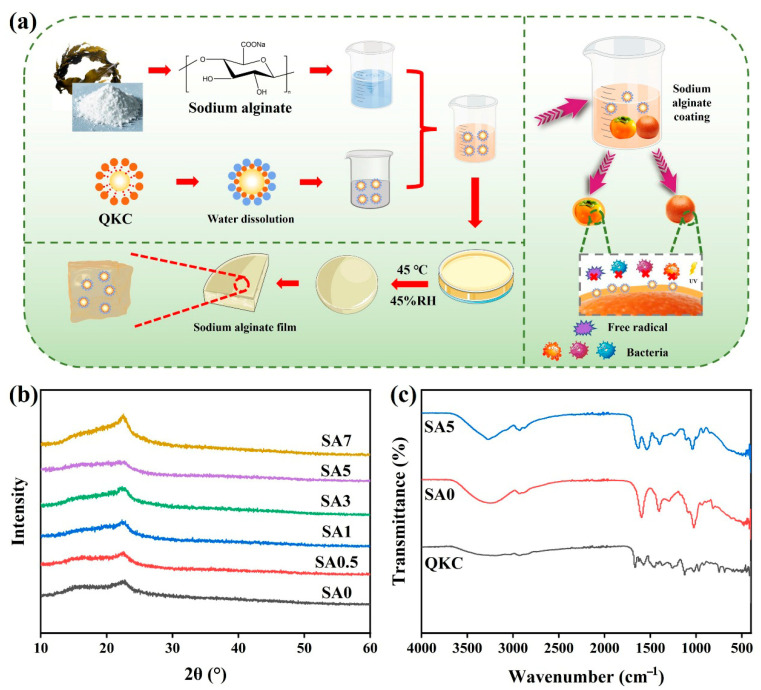
Schematic diagram of the preparation process of SA coating (**a**); XRD patterns of SA coating (**b**); FTIR spectra of QKC, SA0 and SA5 (**c**).

First, a stock SA solution (1.0 g SA and 0.3 g glycerol in 99 mL water) was prepared. For each film, 40 mL of the stock solution (corresponding to 0.40 g SA) was mixed with the desired amount of QKC, as summarized in Table 1. To eliminate entrapped air, the mixture was subjected to ultrasonication for 10 min. Subsequently, the solution was cast into plastic Petri dishes and dried in a climate chamber at 45 °C and 45% RH for 48 h to yield the final coatings. The comprehensive fabrication workflow is illustrated in Figure 1a.

### 2.3. Experimental Method

#### 2.3.1. Structural Analysis

The crystal structure of SA coating was analyzed by X-ray diffraction (Rigaku, D/MAX 2500V, Tokyo, Japan) at a voltage of 20 kV and 30 mA. The chemical structure of QKC, SA0, SA5 were analyzed by Fourier transform infrared spectroscopy (Shimadzu, IRTracer-100, Kyoto, Japan) in the wave-number range of 4000–650 cm^−1^. X-ray photoelectron spectroscopy spectra of QKC, SA0, SA5 were examined by the X-ray photoelectron spectrometer (Thermo Scientific K-Alpha, Waltham, MA, America). Thermogravimetric analysis TGA (Netzsch STA2500, Selb, German) from 30 °C to 700 °C was performed at a heating rate of 10 °C/min under N_2_.

#### 2.3.2. Microstructural Analysis

The surface morphology of the SA coatings was characterized using an in situ SEM nanomechanical properties manipulation system (Carl Zeiss Sigma 300, Oberkochen, Germany) and an atomic force microscope (Bruker, Billerica, MA, USA).

#### 2.3.3. Physical and Mechanical Properties

The color parameters of the SA coating were determined using the automatic color difference analyzer (CA-210, MACKRY, Shenzhen, China). The absorbance and transmittance of the SA coating were evaluated using a UV–Vis spectrophotometer (YuanYuan analysis instrument, Shanghai, China) in the range of 200–800 nm. The SA coating was cut into 25 mm × 2 mm samples with dumbbell-shaped cutters. The tensile strength (TS) and elongation at break (EAB) of the coating were determined using a microcomputer-controlled electronic universal testing machine. The original gauge length was 15 mm, and the tensile speed was 2 mm/min.

#### 2.3.4. Water Barrier Performance

##### Water Vapor Permeability

2.0 g of anhydrous calcium chloride was put into a glass bottle [8]. SA coating was cut into the appropriate circle, which covered the mouth of the bottle with adhesive. The bottle was put into a drying pot, which was filled with the saturated Mg(NO_3_)_2_ solution, controlling the humidity of the drying pot being 80%, and the weight change was measured for 12 h. The water vapor permeability (WVP) of SA coating was calculated according to Equation (1):

(1) $$WVP=\frac{\Delta W\times d}{T\times A\times \Delta P}$$

where WVP (g·m^−1^·h^−1^·Pa^−1^) is the water vapor permeability, ΔW (g) is the weight change in the glass bottle, d (m) is the thickness of the SA coating, T (h) is the time, A (m^2^) is the area available for water vapor transfer, and ΔP (kPa) is the water vapor pressure difference on both sides of the SA coating.

##### Moisture Content

SA coating was placed in the oven and dried until completely dry at a temperature of 105 °C. The weight change in the coating before and after was measured [25]. The moisture content (MC) of SA coating was calculated according to Equation (2):

(2) $$MC=\frac{m_{1}-m_{2}}{m_{1}}\times 100\%$$

where MC (%) is the moisture content; m_1_ (g) is the weight of the SA coating before drying; and m_2_ (g) is the weight of the SA coating after drying.

##### Water Contact Angle

The water contact angle (WCA) was measured with the contact angle measuring instrument (JCY-1, ShangHai FangRui Instrument, Foshan, China).

#### 2.3.5. Antioxidant Activity

DPPH was dissolved in ethanol to prepare a 0.1 mmol/L DPPH solution [26]. 1 mL SA coating solution, which was diluted five times, was evenly mixed with 4 mL DPPH solution at room temperature without light for 30 min. The absorbance value of the mixed solution at 517 nm named A_sample_ was determined using an ultraviolet spectrophotometer, and deionized water was used as the control group named A_control_. The DPPH scavenging activity of SA coating was calculated according to Equation (3):

(3) $$The\;DPPH\;scavenging\;activity\;(\%)=\frac{A_{control}-A_{sample}}{A_{control}}\times 100\%$$

where A_sample_ is the absorbance of the sample and A_control_ is the absorbance of the control sample under the same experimental conditions.

ABTS was dissolved in methanol to prepare a 7.4 mmol/L ABTS solution, while potassium persulfate was dissolved in deionized water to prepare a 7.4 mmol/L solution [26]. ABTS solution and potassium persulfate solution were mixed evenly. The absorbance of the mixture was adjusted to 0.7 ± 0.05 at 734 nm. 1 mL SA coating solution was evenly mixed with 4 mL ABTS solution at room temperature without light for 10 min. The absorbance value of the mixed solution named A_sample_ was determined, and deionized water was used as the control group named A_control_. The ABTS scavenging activity of SA coating was calculated according to Equation (4):

(4) $$The\;\mathrm{ABTS}\;scavenging\;activity\;(\%)\;=\frac{A_{control}-A_{sample}}{A_{\mathrm{control}}}\times 100\%$$

where A_sample_ is the absorbance of the sample and A_control_ is the absorbance of the control sample under the same experimental conditions.

#### 2.3.6. Antibacterial Activity

The antibacterial activities of SA coating were performed with the AGAR diffusion method [27]. Briefly, 0.1 mL of bacterial suspension (*S. aureus* or *E. coli*, 10^7^ CFU/mL) was surface-plated onto Muller-Hinton agar (MHA) plates. Sterile Oxford cups were positioned on the agar surface, and each was filled with 0.2 mL of the coating solution. Following incubation at 37 °C for 24 h, the antibacterial activity was quantified by measuring the diameter of the inhibition zones.

Additionally, the flat coating technique was employed to further assess antibacterial performance. In this assay, MHA plates were inoculated with 0.1 mL of a diluted bacterial suspension (10^5^ CFU/mL). Subsequently, 0.2 mL of the coating solution was uniformly spread over the inoculated agar surface. After a 24 h incubation period at 37 °C, surviving colonies were enumerated. The antibacterial efficacy was expressed as the bacterial mortality rate (%), calculated by comparing the colony counts of the experimental groups against the SA0 control.

#### 2.3.7. Cytotoxicity Experiment

Cytotoxicity of SA coating was detected by MTT assay, and L929 mouse fibroblasts (NCTC clone 929) were used as representative cells [8]. According to the ratio of MEM medium to fetal bovine serum of 9:1, the cell medium was prepared, and the cell suspension of L929 mouse fibroblasts was prepared with trypsin and cultured in a 5% CO_2_ incubator at 37 °C. The control group was added with 100 μL/well complete culture medium, and the experimental group was added with 100 μL/well SA coating solution. 6 multiple wells were made in each treatment group. L929 cells at logarithmic growth stage were taken for cell count, cell concentration was adjusted, and the cells were inoculated into 96-well plates according to 5 × 10^3^/well, cultured in a constant temperature incubator at 37 °C with 5% CO_2_ for 48 h. Remove the medium, clean each hole three times with PBS solution, add 100 μL of medium containing 0.5 mg/mL MTT to each hole, 5% CO_2_, and culture in a constant temperature incubator at 37 °C for 4 h. Discard the supernatant and add 100 μL DMSO per well. After gently shaking for 10 min, the absorbance value at 570 nm wavelength was detected. Cell survival rate (SR) was calculated according to Equation (5):

(5) $$SR(\%)=\frac{A_{Sample}}{A_{control}}\times 100\%$$

where A_sample_ is the absorbance of the sample and A_control_ is the absorbance of the control sample under the same experimental conditions.

#### 2.3.8. Preservation of Persimmon and Tangerine (Coating Application)

Persimmons and tangerines of uniform size, shape, and maturity were selected and washed with deionized water. The fruits were surface-sterilized by immersion in 0.3% (*v*/*v*) sodium hypochlorite solution for 5 min, rinsed with sterile water, and air-dried. The fruits were then randomly divided into two groups. The experimental group was immersed in the SA5 coating solution for 30 s, removed, and air-dried. This coating process was repeated three times to ensure a uniform layer. The control group was treated with deionized water under the same conditions. All samples were stored under ambient laboratory conditions (25 ± 2 °C and 50 ± 5% RH). Quality parameters were measured at regular intervals (every 2 days for tangerines and every 4 days for persimmons). Weight Loss Rate: Calculated gravimetrically by comparing the weight of the fruit on the sampling day with its initial weight. Surface Hardness: Measured using a Shore A hardness tester. The probe was pressed vertically into the fruit surface until it penetrated the skin, and the peak value was recorded (expressed in HSD). pH Value: Determined according to the method of Nkede [28] et al. with slight modifications. A specific amount of fruit pulp was homogenized with deionized water, and the pH of the filtrate was measured using a digital pH meter.

#### 2.3.9. Preparation of Sodium Alginate Gel Beads and Blueberry Preservation

To prepare SQCa gel beads, 2.0 g of SA was dissolved in 98 mL of deionized water, followed by the addition of 50 mL of QKC emulsion (2 wt%) under continuous stirring. The mixture was added dropwise into a 5 wt% calcium chloride (CaCl_2_) solution using a peristaltic pump to form cross-linked beads. The resulting SQCa beads were rinsed multiple times with deionized water.

Blueberries of uniform size and maturity were selected, washed with deionized water, and dried. For the preservation test, 5 blueberries were placed in a beaker. In the experimental group, 20 SQCa gel beads were added to the beaker, which was then sealed with cling film. The control group contained blueberries without gel beads. All samples were stored at room temperature.

Titratable Acidity (TA): TA was determined by titration. 5–10 g of blueberry pulp was homogenized and filtered. 20 mL of the filtrate was mixed with 1 drop of 1% phenolphthalein indicator and titrated with 0.04 mol/L NaOH solution until a faint pink color persisted for 30 s. The TA content was calculated according to Equation (6):

(6) $$Titratable\;acidity(\%)=\frac{(V_{1}-V_{0})c\times f\times V}{V_{2}\times m}\times 100\%$$

where V_1_ (mL) is the volume of NaOH solution consumed by titrating filtrate; V_0_ (mL) is the volume of NaOH solution consumed by titrating deionized water; V (mL) is the total volume of sample extract; V_2_ (mL) is the volume of titration filtrate; c (mol/L) is the concentration of NaOH solution; f (g/mmol) is the conversion coefficient, f = 0.067; and m (g) is the sample quality.

### 2.4. Statistical Analysis

All experiments were performed in triplicate, and the data are expressed as the mean ± standard deviation (SD). Statistical significance was determined at *p* < 0.05.

## 3. Results and Discussion

### 3.1. Structural Analysis of SA Coating

To elucidate the formation mechanism and structural interactions within the composite coatings (fabrication process illustrated in Figure 1a), the crystalline and chemical structures were systematically characterized using XRD (Figure 1b) and FTIR (Figure 1c), respectively. As shown in the XRD patterns (Figure 1b), a weak diffraction peak at 2θ = 22.60° is associated with the largely amorphous nature of sodium alginate. With increasing QKC loading, the characteristic SA diffraction band progressively broadened, indicating reduced crystallinity within the coatings. SA and QKC interact via electrostatic attraction and hydrogen bonding, forming an amorphous complex and thereby decreasing the internal crystallinity of the SA coatings [29]. At the highest loading, SA7, the diffraction features became sharper again, suggesting partial phase separation and local ordering due to limited compatibility between excess QKC and the alginate matrix.

FTIR spectra of QKC, SA0, and SA5 are presented in Figure 1c. The broad band at 3270 cm^−1^ corresponds to O-H stretching [30]. Lower intensity of this band in QKC reflects consumption of phenolic O-H groups during lignin quaternization. Relative to SA0, the O-H band intensity in SA5 increased, consistent with hydrogen-bonding interactions between SA and QKC. The band near 2930 cm^−1^ is assigned to C-H stretching; bands at 1595 and 1400 cm^−1^ correspond to asymmetric and symmetric carboxylate stretching [31]; the band near 1025 cm^−1^ arises from C-O stretching [32]. The overall spectral profiles of SA0 and SA5 were similar, indicating that SA and QKC associate predominantly through physical interactions (hydrogen bonding and van der Waals forces) without evidence of new covalent bond formation. No new peaks or significant shifts indicative of chemical derivatization were observed.

**Figure 2:**
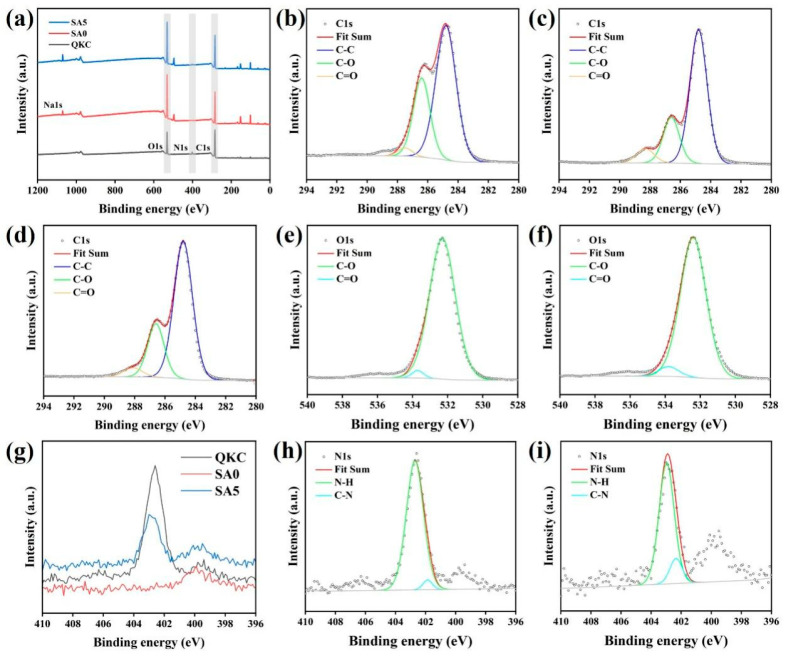
The XPS full survey spectra of QKC, SA0 and SA5 (**a**); high-resolution C1s spectrum of QKC (**b**); and SA0 (**c**); and SA5 (**d**); high-resolution O1s spectrum of SA0 (**e**); and SA5 (**f**); high-resolution N1s spectrum of QKC, SA0 and SA5 (**g**); and QKC (**h**); and SA5 (**i**).

XPS was conducted to probe the chemical composition of QKC, SA0, and SA5 (Figure 2). Survey spectra showed signals at binding energies of 167.31 eV, 284.43 eV, 401.83 eV, and 531.76 eV, attributable to S2p, C1s, N1s, and O1s, respectively. In SA0 and SA5, an additional peak at 1070.83 eV corresponds to Na1s from SA. In the high-resolution C1s spectra, components near 284.8, 286.4, and 287.5 eV are assigned to C-C, C-O, and C=O, respectively, and are present in QKC, SA0, and SA5. In the O1s spectrum of SA0, peaks at 532.3 and 533.7 eV are associated with C-O and C=O; upon QKC addition, the C=O contribution increased, consistent with cinnamaldehyde. SA0 showed no N1s signal, as alginate lacks nitrogen. Relative to QKC, the N1s component in SA5 shifted to higher binding energy, reflecting stronger electrostatic interactions between quaternary ammonium groups and alginate carboxylates and altered electron density. The quaternary ammonium N+ component increased from 402.71 eV to 402.97 eV, consistent with an electrostatic and hydrogen-bonding network between QKC and SA.

**Figure 3:**
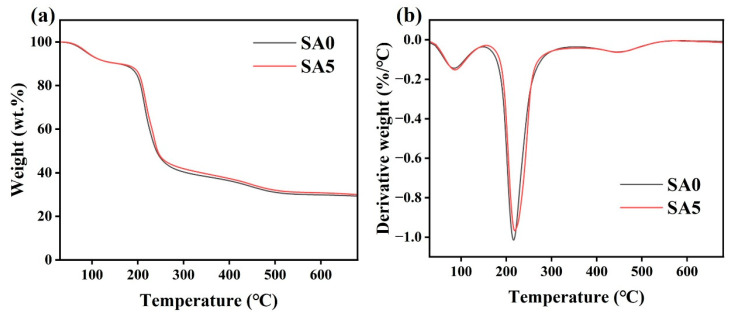
TGA curves of SA0 and SA5 (**a**); DTG curves of SA0 and SA5 (**b**).

DTG and TGA analyses of SA0 and SA5 are shown in Figure 3. Mass loss proceeds in three stages. Stage I (50–150 °C) corresponds to evaporation of free water [33]. Stage II (150–340 °C) is primarily attributable to glycerol volatilization and decomposition and scission of the sodium alginate backbone [34]. In SA0, the maximum degradation rate occurred at 215 °C; with QKC addition, the corresponding peak for SA5 appeared at 220 °C and its magnitude decreased, indicating a denser interaction network between QKC and SA, enhanced intermolecular forces, and improved thermal stability. Stage III (>340 °C) involves continued breakdown of C-C and C-H bonds with evolution of volatile products [35]. These trends are consistent with reduced chain mobility and stronger intermolecular interactions upon QKC incorporation.

### 3.2. Microstructural Analysis of SA Coating

**Figure 4:**
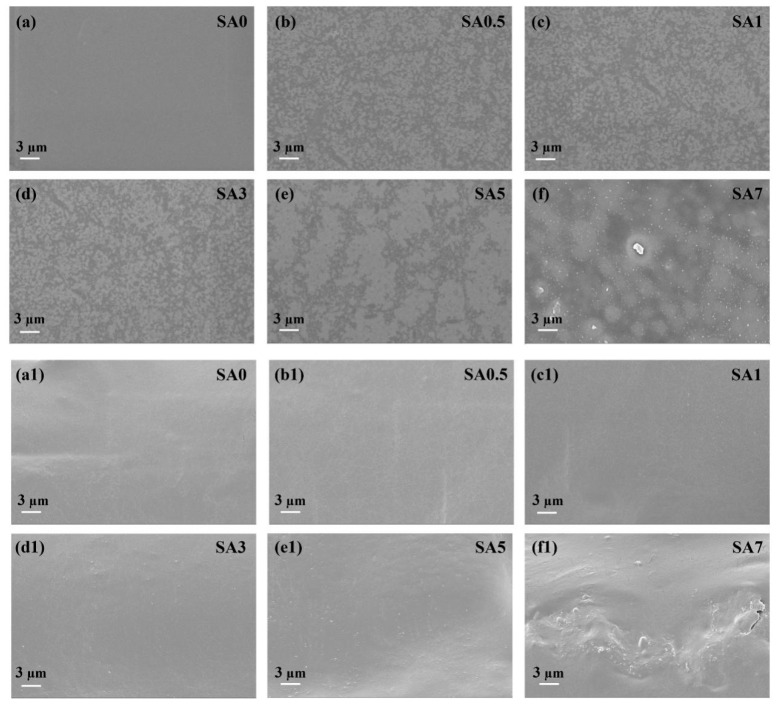
Surface SEM image of SA coating (**a**–**f**); section SEM image of SA coating (**a1**–**f1**).

The surface and cross-sectional SEM images of SA coatings are shown in Figure 4. The surface of SA0 was uniform and smooth, without pores or cracks, highlighting the excellent film-forming capability of SA. Upon QKC addition, white dot-like features appeared on the surface and were uniformly distributed, consistent with mechanical reinforcement. In SA5, QKC exhibited white block-like aggregates while the surface remained smooth. At 7 percent loading, block-like protrusions were evident, indicating phase segregation and compromised surface morphology due to limited SA-QKC compatibility. In cross-section, the grooves observed in SA0 suggest a hydrogen-bonded network between glycerol and alginate that compacts the internal structure. With QKC addition, an additional hydrogen-bonded network formed between QKC and alginate, further densifying the interior. When QKC was excessive, unincorporated QKC deposited within the matrix, disrupting continuity and internal alignment, consistent with incomplete compatibility.

AFM analysis (Figure S1) was performed to further quantify the surface topography. The average roughness (Ra) values for SA0, SA0.5, SA1, SA3, SA5, and SA7 were 126.64, 130.05, 144.41, 146.17, 173.75, and 197.45 nm, respectively. Similarly, the root-mean-square roughness (Rq) values were 237.45, 239.56, 259.96, 267.65, 282.23, and 313.91 nm, respectively.

Both Ra and Rq metrics demonstrate a clear, dose-dependent upward trend, confirming that the incorporation of QKC particles progressively increases the surface roughness. This is attributed to the QKC particles being embedded within the SA network and partially protruding from the surface, creating a more complex topography. These quantitative findings are in strong agreement with the qualitative observations from the SEM surface images (Figure 4), which also showed increased particle aggregation and surface features at higher QKC loadings (e.g., SA5 and SA7), indicative of partial phase segregation.

### 3.3. Physical and Mechanical Properties

**Figure 5:**
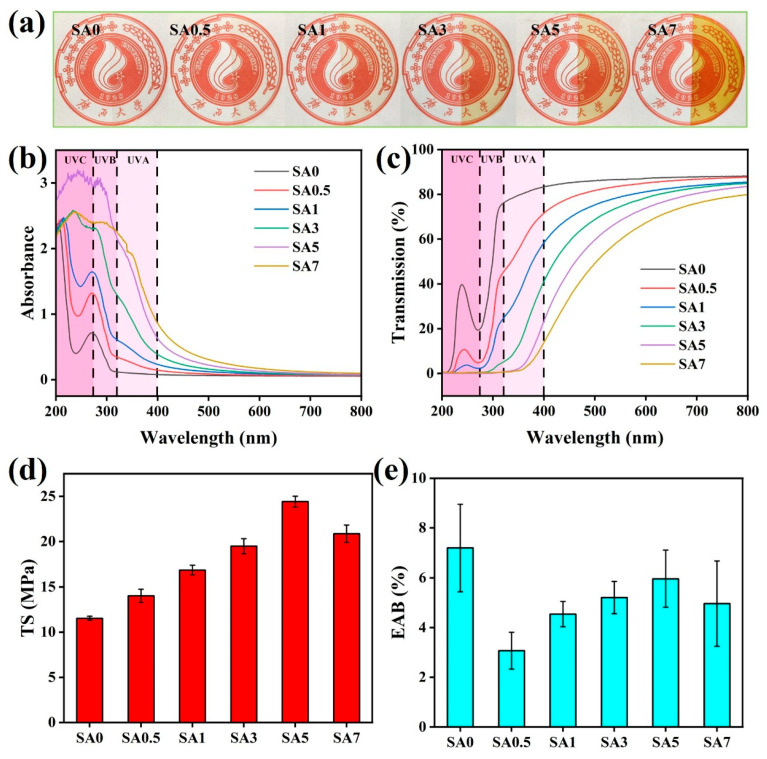
Macro image of SA coating (**a**); UV absorption spectrum of SA coating (**b**); UV transmittance spectra of SA coating (**c**); tensile strength of SA coating (**d**); elongation at break of SA coating (**e**).

Color parameters were measured using a colorimeter, and results are provided in Table 2. The L* value of SA0 was highest, while a* and b* were lowest, indicating a nearly colorless, transparent film, consistent with the photographic image in Figure 5a. With increasing QKC, L* decreased and a* and b* increased, reflecting progressive reddish-yellow coloration and reduced brightness. This behavior mainly arises from chromophoric phenolic and carbonyl moieties in lignin. The ΔE* of the SA coatings increased with QKC content, mirroring the visible color change in Figure 5a. The background beneath the films remained clearly visible; thus, when applied to fruit, the coatings would not hinder visual inspection and are compatible with practical use. All samples maintained high transparency in the visible range, suitable for surface observation during storage.

UV absorption and transmittance spectra are shown in Figure 5b,c. SA0 exhibited poor UV-blocking efficacy, with high transmittance across the UV range (80.9 percent at 365 nm). QKC markedly enhanced UV shielding, which increased with loading; SA5 showed 5.2 percent transmittance at 365 nm, which is attributed to lignin in QKC. Under UV irradiation, phenylpropanoid units in lignin can form quinonoid structures that attenuate UV, and intrinsic phenolic and carbonyl groups further strengthen absorption [36]. QKC incorporation imparts effective UVA (315–400 nm) protection to the SA coatings.

The mechanical properties of the SA coatings were evaluated (Figure 5d,e). For SA0, TS was 11.53 MPa and the elongation at break (EAB) was 7.20%. Upon incorporation of QKC, TS increased in a dose-dependent manner. The incorporation of QKC significantly reinforced the SA matrix, with TS increasing from 11.53 MPa to 24.42 MPa (SA5). This reinforcement effect (111.8% increase) is superior to many reported SA-based composite films. For instance, Nkede [28] et al. reported that the direct incorporation of *Helichrysum italicum* essential oil into alginate films decreased the TS from 16.19 MPa to 13.25 MPa due to the plasticizing effect of oil droplets that disrupt the polymer network. Similarly, Deng [37] et al. found that adding whey protein isolate to SA films only improved TS from 7.33 MPa to 10.08 MPa. In contrast, our QKC particles acted as multifunctional fillers, forming strong electrostatic and hydrogen-bonding crosslinks, similar to the reinforcement observed by Wu [38] et al., where *Dendrobium officinale* polysaccharides increased the TS of SA films to 20.77 MPa. The ultra-high strength of SA5 suggests that the quaternized lignin skeleton effectively anchors the cinnamaldehyde, avoiding the structural weakening often seen with direct essential oil addition. TS of the SA coating kept increasing, and TS of SA5 coating rose to 24.42 MPa, which was 111.80% higher than that of SA0 coating. When QKC was excessive (7%), phase incompatibility disrupted chain ordering and weakened the network, leading to a reduction in TS.

Conversely, the EAB (Figure 5e) decreased significantly upon the initial addition of QKC (SA0.5) and never recovered to the level of the neat SA0 film. This is a classic behavior for particle-reinforced composites. The QKC particles (acting as rigid fillers) and the enhanced electrostatic/hydrogen bonding network increased the film’s stiffness and strength (TS), but simultaneously restricted the mobility and extensibility of the SA polymer chains, leading to a more brittle film with lower EAB. The severe drop in EAB at 7% QKC is consistent with the TS data, where particle aggregation (phase incompatibility) created defect points that promoted premature fracture.

### 3.4. Water Barrier Performance

**Figure 6:**
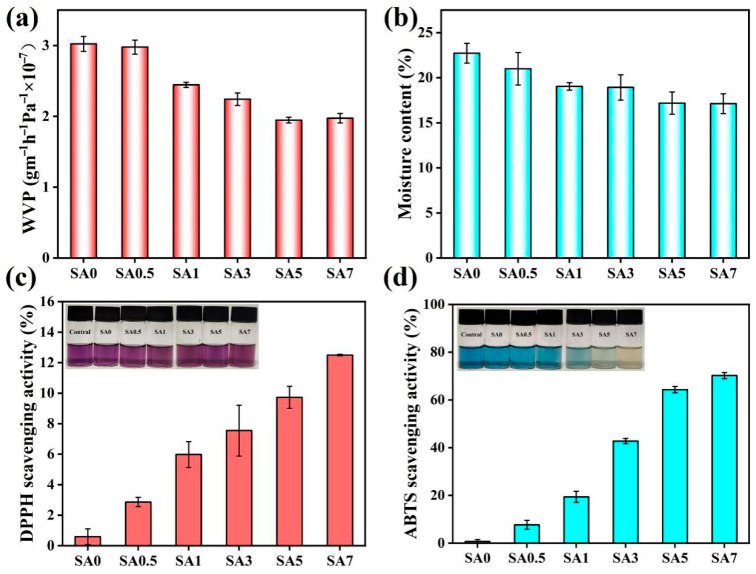
Water vapor permeability of SA coating (**a**); moisture content of SA coating (**b**); DPPH free radical scavenging rate of SA coating (**c**); ABTS free radical scavenging rate of SA coating (**d**).

WVP was measured (Figure 6a). As QKC content increased, WVP decreased and then slightly increased, with a minimum at 5% QKC. QKC-SA hydrogen-bonding and electrostatic complexation strengthened the network and hindered water diffusion. Additionally, the hydrophobic cinnamaldehyde component impeded water transport, further enhancing the barrier performance. At 5% QKC, WVP dropped to the lowest value, from 3.02 × 10^−8^ to 1.95 × 10^−8^ g·m^−1^·h^−1^·Pa^−1^. This barrier improvement (35.4% reduction) is consistent with recent findings on particle-reinforced alginate systems. Liu [39] et al. reported that the inclusion complex of Magnoliae essential oil with cyclodextrin reduced the water vapor transmission of SA coatings by creating a tortuous path for water molecules. Furthermore, our results outperformed some non-crosslinked systems; for example, Abouzeid [40] et al. reported that non-crosslinked sweet potato peel nanomaterial/SA films exhibited significantly higher oxygen and water vapor permeability compared to crosslinked counterparts. The hydrophobic aromatic rings of lignin and the aldehyde groups of CIN in our QKC particles likely contributed to the increased tortuosity and hydrophobicity, effectively blocking moisture transport. At 7% QKC, partial phase separation and particle aggregation introduced microvoids and disrupted continuity, which facilitated vapor transport and increased WVP.

Static water contact angles (CA) are shown in Figure S2. The CA of SA coating were 60.13°, 61.93°, 63.87°, 65.23°, 66.83° and 68.57°, respectively, demonstrating a clear and dose-dependent increase in surface hydrophobicity with higher QKC loading. Interfacial complexation between QKC and SA likely consumed accessible hydroxyls, lowering surface polarity. Moreover, cinnamaldehyde enriches hydrophobic domains at the surface, further increasing CA.

Moisture content (MC) is reported in Figure 6b. Relative to SA0, MC decreased progressively as QKC increased. Enhanced QKC-SA hydrogen bonding reduced the availability of sites for water-polymer interactions. Hydrophobic domains introduced by cinnamaldehyde further limited water uptake. Lower MC is expected to improve barrier performance and contribute to better fruit preservation.

Although the elongation at break (EAB) and water vapor barrier performance of the composite coatings are limited compared to some hydrophobic synthetic polymers, the SA5 coating offers a superior balance of antibacterial, antioxidant, and UV-shielding properties. These functional attributes are critical for inhibiting microbial growth and delaying oxidation-induced senescence, as evidenced by the effective preservation of persimmons and tangerines. Furthermore, the inherent hydrophilicity of the alginate matrix serves as a practical advantage for edible coatings: unlike hydrophobic wax coatings that can be difficult to remove, the SA5 coating can be easily washed off with water prior to consumption, ensuring food safety and consumer convenience.

### 3.5. Antioxidant Ability

DPPH and ABTS assays were used to assess antioxidant activity (Figure 6c,d). SA0 exhibited negligible DPPH scavenging, consistent with the absence of intrinsic antioxidant groups in SA. Increasing QKC content progressively enhanced radical scavenging. Phenolic hydroxyls in lignin are the primary contributors to antioxidant activity via hydrogen atom and electron transfer.

A significant difference in scavenging capacity was observed between the two assays. The ABTS method (Figure 6d) yielded substantially higher antioxidant values, reaching a maximum of 70.22% for SA7, compared to the DPPH method (Figure 6c, SA7 at ~12.5%). This discrepancy is likely attributed to the assay solvents and mechanisms. SA tends to gel in the ethanol used for the DPPH assay, limiting reagent diffusion; although samples were diluted, residual gelation likely reduced the apparent DPPH scavenging activity. The ABTS assay, being aqueous, did not have this limitation. Therefore, while both assays confirm a clear dose-dependent antioxidant trend, the ABTS result (70.22%) is considered more representative of the coating’s intrinsic radical scavenging potential.

### 3.6. Antimicrobial Ability

**Figure 7:**
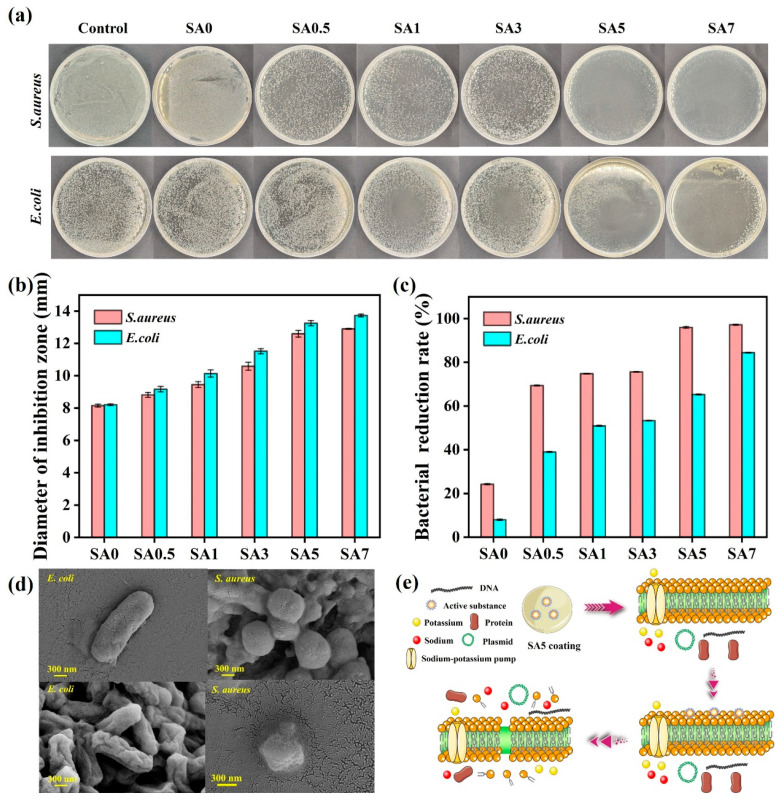
Antibacterial effect of SA coating against *S. aureus* and *E. coli* with Flat Coating method (**a**); the inhibition zone diameters of SA coating against *S. aureus* and *E. coli* (**b**); the inhibition rate of SA coating against *S. aureus* and *E. coli* (**c**); SEM images of *S. aureus* and *E. coli* before and after being treated with SA5 coating (**d**); antibacterial mechanism diagram of SA5 coating (**e**).

Antibacterial activity against *S. aureus* and *E. coli* was assessed using the Oxford cup and plate-coating methods (Figure 7). Inhibition zone diameters increased with QKC loading: for *S. aureus*, 8.15, 8.81, 9.45, 10.59, 12.60, and 12.90 mm for SA0–SA7, respectively; for *E. coli*, 8.20, 9.16, 10.14, 11.52, 13.25, and 13.73 mm, respectively. The antibacterial performance of SA coating determined by the Flat Coating method showed the same result. Overall, antibacterial efficacy correlated positively with QKC content. For SA7, inhibition rates remained ≥97% for *S. aureus* and ≥84% for *E. coli*, demonstrating excellent antibacterial performance. The antibacterial experiment with Flat Coating method showed that SA coating presented a better antibacterial effect on *S. aureus*. The *S. aureus* is a Gram-positive bacterium whose phosphate-rich cell wall confers a strong negative surface charge. Positively charged QKC particles readily interact with the anionic cell envelope, promoting contact killing and membrane destabilization. By contrast, Gram-negative *E. coli* has an outer membrane that reduces penetration of cationic agents, attenuating activity. SEM imaging before and after SA5 treatment showed collapsed, wrinkled morphologies consistent with membrane damage and loss of turgor. Mechanistically, quaternary ammonium groups mediate electrostatic attraction and membrane disruption, while cinnamaldehyde perturbs lipid organization and increases permeability, leading to leakage of intracellular contents and cell death.

### 3.7. Cytotoxicity Experiment

Cytotoxicity was assessed by the MTT assay using L929 mouse fibroblasts after 48 h exposure (Figure S3). The cell survival rates after treatment with SA0, SA0.5, SA1, SA3, SA5 and SA7 coating were 96.70%, 91.22%, 89.36%, 87.13%, 86.13% and 85.01%, respectively. All cell survival rates exceeded 80%, and the mouse fibroblasts showed high viability. The L929 mouse fibroblasts without drug treatment were used as the control group. Before the experiment, the growth of L929 mouse fibroblasts was sparse and the number was small. After 48 h of co-culture, the mouse fibroblasts in both the control group and the SA5 group grew densely, and there was no phenomenon of large-scale cell death. These results, with all samples exhibiting cell survival rates well above the 80% cytotoxicity threshold (according to ISO 10993-5, 2009 [8]), indicate that the QKC particles do not induce significant cytotoxicity at the tested concentrations. The SA5 coating maintained high biocompatibility (cell survival rate > 86% for L929 fibroblasts), which is a critical advantage for food packaging safety. In comparison, some essential oil-loaded films require high concentrations to achieve antibacterial efficacy, often compromising biocompatibility. Zhang [41] et al. developed chitosan/PVA films loaded with cinnamaldehyde-zein nanoparticles, which showed strong antibacterial activity but did not explicitly evaluate cytotoxicity in relation to the particle concentration. Our study confirms that encapsulating CIN within quaternized lignin (QKC) mitigates the potential cytotoxicity of high-dose free cinnamaldehyde while retaining antimicrobial efficacy. This aligns with findings by Yu [42] et al., who demonstrated that natural polymer-based hydrogels (SA/Nanocellulose) could achieve excellent antibacterial properties without compromising safety. The slight reduction in cell viability at higher QKC loadings (SA7) is typical for quaternary ammonium compounds but remains well within acceptable safety limits (>80%) for food contact materials.

### 3.8. Application in Persimmon and Tangerine Preservation (Coating Method)

**Figure 8:**
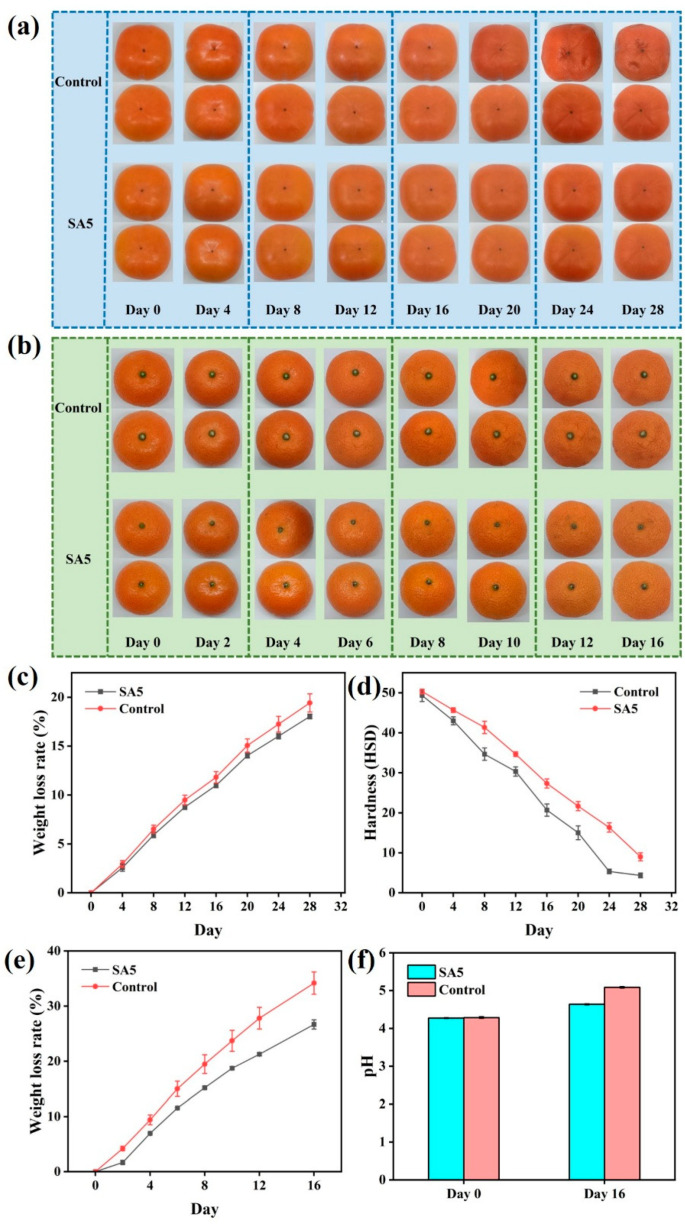
The effect of persimmon preservation with SA5 coating (**a**); the effect of tangerine preservation with SA5 coating (**b**); weight loss rate of persimmon between the experimental group and the control group (**c**); hardness of persimmon between the experimental group and the control group (**d**); weight loss rate of tangerine between the experimental group and the control group (**e**); the pH value of tangerine between the experimental group and the control group (**f**).

Persimmons and tangerines were selected as model fruits to evaluate the preservation efficacy, and the results are shown in Figure 8. At the beginning of the experiment, the surface of the persimmon was smooth and uniform, maintaining a tangerine-red color. As the experimental time extended, the color of the persimmon skin gradually deepened. After 20 days of storage, the skin began to soften and collapse inward, and the wrinkles on the surface of the persimmon could be clearly observed. With SA5 coating treatment, no wrinkles or collapse occurred on the surface of the persimmon. After 24 days of storage, the color of the persimmon’s skin had just begun to change significantly. Compared with the SA5-coated group, the weight-loss rate of persimmon in the control group was higher and increased more rapidly (Figure 8c). During storage, the change in weight loss rate was mainly caused by transpiration water loss and the consumption of nutrients. The SA5 coating formed a barrier on the surface of persimmon, reducing the water exchange between persimmon and the external environment and lowering the water loss of persimmons themselves. The SA5 coating also reduced the oxygen exchange between persimmon and the external environment, lowered the respiration of persimmon, and decreased the loss of nutrients such as sugar and organic acids as respiration substrates, thereby reducing the weight loss rate of persimmon. The respiration of persimmon was inhibited by SA5 coating, the decomposition rate of soluble sugars and other organic macromolecules was reduced, nutrients were retained, persimmon was less likely to wrinkle, and the decrease in epidermal hardness was relatively slow.

During the experiment, after 6 days of storage, the skin of the tangerine in the control group began to shrink. After 8 days of storage, a significant inward collapse was observed. However, the tangerine in the experimental group, which had been treated with SA5 coating, did not show any obvious collapse throughout the experiment. Only after 8 days of storage did they start to shrink. The skin of the tangerine in the experimental group treated with SA5 coating would form a dense coating. The coating could reduce the exchange of moisture and oxygen between the tangerine and the external environment, lower water evaporation and respiration, and slow down the weight loss of the tangerine in the experimental group. With the extension of the experimental time, the pH value of the tangerine continuously increased, and the sour taste of the tangerine gradually decreased. Tangerine sourness primarily derives from organic acids, which were readily utilized as respiratory substrates and thus declined during storage, leading to a rise in pH (Figure 8f). Accordingly, the SA5 coating attenuated the rate of pH increased by moderating respiration and conserving organic acids.

Overall, the SA5 coating significantly extended the shelf life of persimmons and tangerines. This preservation efficacy is comparable to or better than recent advanced alginate coatings. Wu [43] et al. demonstrated that alginate coatings with pear chitinase reduced the weight loss of cherry tomatoes to approximately 4.43% after 21 days. Similarly, Lei [44] et al. reported that SA-based coatings modified with selenium nanoparticles effectively delayed the yellowing and weight loss of fresh-cut bamboo shoots. Our SA/QKC coating not only serves as a physical barrier but also provides active antimicrobial protection, similar to the mixed LAB culture-loaded alginate coatings reported by Fernandes [45] et al., which effectively controlled anthracnose in guava and mango. The dual mechanism of barrier protection and active release of CIN from the QKC/SA matrix explains the superior preservation performance observed in our study.

However, there are certain limitations in the current study. While physical quality indicators (weight loss, firmness) and visual decay were effectively monitored, nutritional parameters such as soluble solids and total phenolic content were not measured. Additionally, although in vitro tests confirmed strong antibacterial activity, direct quantitative microbial counts (CFU/g) on the fruit surface were not performed. We plan to address these aspects in follow-up research by including metabolic analysis and in situ microbial monitoring to provide a more holistic evaluation of the preservation mechanism.

### 3.9. Application in Blueberry Preservation (Gel Bead Method)

**Figure 9:**
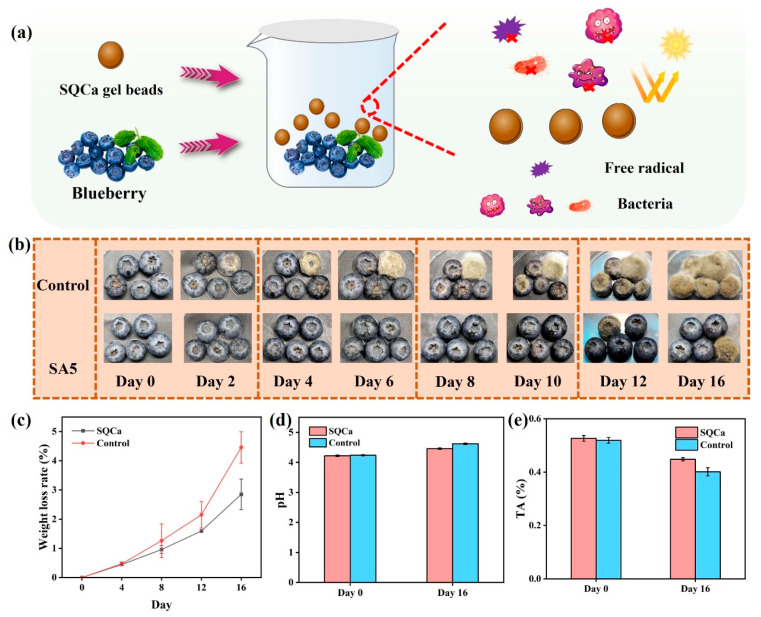
Schematic diagram of blueberry preservation with SQCa gel beads (**a**); the effect of blueberry preservation with SQCa gel beads (**b**); weight loss rate of blueberry between the experimental group and the control group (**c**); the pH of blueberry between the experimental group and the control group (**d**); titratable acidity of blueberry between the experimental group and the control group (**e**).

To broaden the application of the SA coating, SQCa gel beads were prepared by cross-linking with Ca^2+^. This format was especially suitable for small fruits for which a direct coating is impractical. Therefore, SQCa gel beads were prepared as a convenient format that did not require a cleaning process. The blueberries were selected as the experimental fruit, and the results are shown in Figure 9. After 2 days of storage, mold could already be observed on the blueberries in the control group. As the experimental time extended, the mold continued to spread. After 10 days of storage, all the blueberries showed mold, and after 16 days of storage, they were all covered with mold. The blueberries in the experimental group showed signs of mold only after being stored for 10 days. After 16 days of storage, only a few blueberries were covered with mold, indicating that SQCa gel beads could effectively inhibit the growth of bacteria and enhance the preservation effect. The flavor of post-harvest blueberries was greatly influenced by titratable acids. The color, taste and storage time of blueberries were all affected by titratable acids. The pH value of blueberry juice could also reflect the overall taste of the blueberry. The pH is part of the titratable acid index. The higher the pH value, the lower the value of titratable acids was generally. As the storage time continued to extend, the maturity of blueberries gradually increased, organic acids were continuously decomposed and consumed to form sugars, and the pH value of the blueberry juice gradually rose. SQCa gel beads possessed antioxidant and antibacterial activities, which could inhibit the growth and reproduction of microorganisms on the surface of blueberries, reduce the rate of oxidation reactions, decrease the number of blueberry cells that die due to microbial damage, lower the consumption of nutrients inside the blueberry fruit, slow down the deceleration rate of organic acids, and extend the preservation time of blueberries. SQCa gel beads could slow down the metabolic process of post-harvest blueberries, reduce the damage of microorganisms to the surface of blueberries, affect the consumption and metabolism of nutrients such as titratable acids, and thereby increase the preservation time of blueberries.

## 4. Conclusions

In this study, quaternary ammonium lignin–cinnamaldehyde composite particles and sodium alginate were cross-linked through electrostatic and hydrogen bonding, and interfacial interactions during particle dispersion enhanced network stability, thereby constructing a composite antibacterial coating with a uniform structure and stable interface. In addition, the coating’s hydrophobicity and barrier properties were significantly improved, with the optimized SA5 formulation achieving a 35.4% reduction in WVP. The SA5 coating demonstrated ultraviolet-blocking ability and exhibited strong antioxidant activity; the highest ABTS free radical scavenging rate (70.22%) was observed for SA7. Most notably, the mechanical properties were significantly enhanced, with the SA5 coating showing a 111.80% increase in TS (from 11.53 MPa to 24.42 MPa) compared to the neat SA film. The inhibition zone diameters against *S. aureus* and *E. coli* reached 12.60 mm and 13.25 mm, respectively. The inhibition rates determined by the Flat Coating method were 96% and 65%, respectively. Preservation experiments with tangerines and persimmons showed that the SA5 coating could slow water loss and nutrient consumption and extend storage life. For blueberries, SQCa gel beads markedly delayed visible mold growth compared with the control.

## Figures and Tables

**Table 1:** Composition of SA coating.

Samples	SA (g)	QKC (g)	QKC Percentage (wt%)
SA0	0.4	0	0
SA0.5	0.4	0.002	0.5
SA1	0.4	0.004	1
SA3	0.4	0.012	3
SA5	0.4	0.020	5
SA7	0.4	0.028	7

**Table 2:** Color difference values of SA coating.

Samples	L*	a*	b*	dE*
SA0	91.95 ± 0.036	4.63 ± 0.059	1.64 ± 0.045	0
SA0.5	91.33 ± 0.006	5.47 ± 0.025	1.92 ± 0.010	1.07 ± 0.025
SA1	90.69 ± 0	6.55 ± 0.006	2.38 ± 0.006	2.41 ± 0.006
SA3	85.28 ± 0.010	16.77 ± 0.035	9.15 ± 0.015	15.76 ± 0.038
SA5	83.29 ± 0.012	21.28 ± 0.010	11.26 ± 0.010	21.09 ± 0.015
SA7	81.84 ± 0.015	24.3 ± 0.029	13.21 ± 0.032	24.96 ± 0.044

## Data Availability

The data presented in this study are available in the article and its associated Supplementary Materials. Further inquiries can be directed to the corresponding authors.

## Supplementary Materials

The following supporting information can be downloaded at https://www.mdpi.com/article/10.3390/foods14244203/s1, Figure S1: Surface topography and roughness parameters of SA coatings; Figure S2: Static water contact angles of SA coatings; Figure S3: Cytotoxicity assessment of SA coatings by MTT assay.
