# Supplementary material for: Sodium Alginate-Based Antibacterial Coatings Reinforced with Quaternized Lignin–Cinnamaldehyde Composite Particles for Fruit Preservation
Source: Foods. 2025 Dec 7;14(24):4203. doi: 10.3390/foods14244203 (PMC12731641; doi:10.3390/foods14244203)
Supplement: Supplementary file 1 [file foods-14-04203-s001.zip › foods-4001278-supplementary.pdf]

Supplementary Materials

Sodium Alginate-Based Antibacterial Coatings Reinforced with Quaternized Lignin–Cinnamaldehyde Composite Particles for Fruit Preservation

Jianshuo Miao [1,2], Yuanrong Lai [2], Yidan Zhang [2], Jiapeng Wei [2], Kehao Fan [2], Ningjing Sun [1,*] and Zhiyong Qin [2,*]

1 College of resources and environment sciences, Baoshan University, Baoshan 678000, China; mjs1790@163.com
2 School of Resources, Environment and Materials, Guangxi University, Nanning 530004, China; yuanronglai@163.com (Y.L.); zhangyidanegg@163.com (Y.Z.); plianasp@163.com (J.W.); fankehaoo@163.com (K.F.)
* Correspondence: sunningjing-bsnc@vip.163.com (N.S.); qinzhiyong@gxu.edu.cn (Z.Q.)

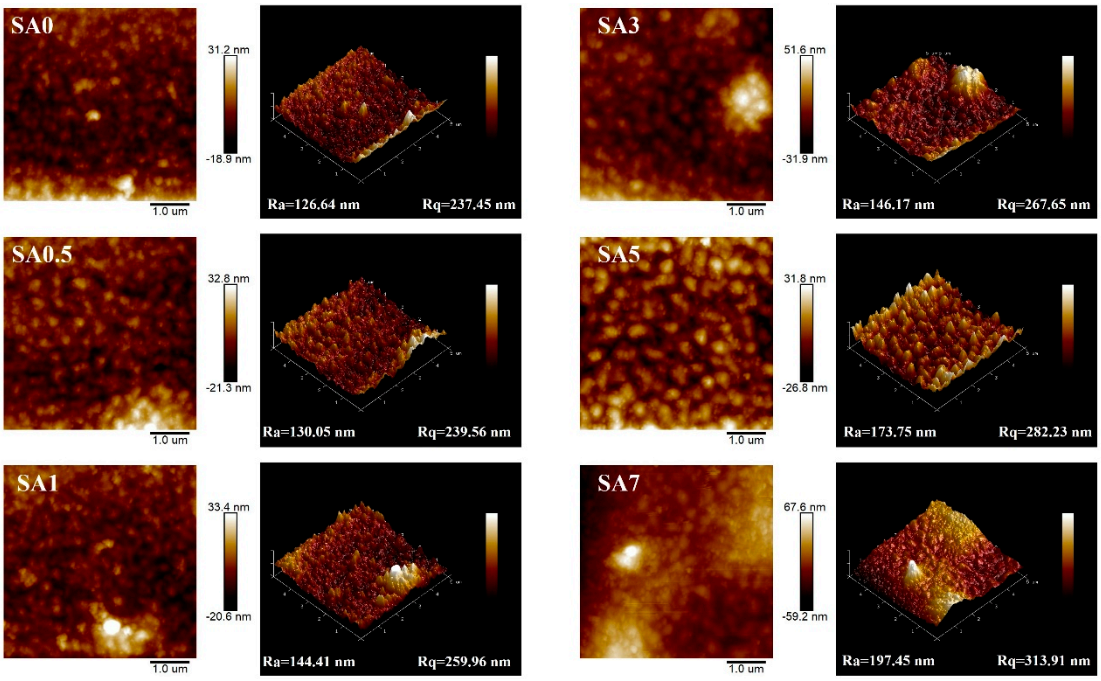

Figure S1. Surface topography of SA coatings. 2D and 3D atomic force microscopy (AFM) images (1.0 μm x 1.0 μm scan area) for SA0, SA0.5, SA1, SA3, SA5, and SA7 films. The corresponding average roughness (Ra) and root-mean-square roughness (Rq) values are indicated for each sample, showing a progressive increase in surface roughness with QKC loading.

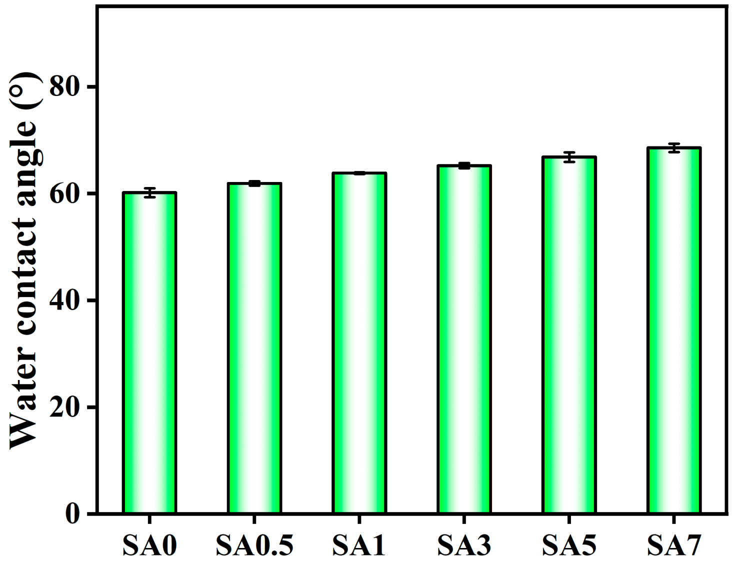

Figure S2. Water contact angle (WCA) measurements of SA coatings. Data shows the static water contact angles for SA0, SA0.5, SA1, SA3, SA5, and SA7 films. The WCA progressively increases with QKC loading, indicating enhanced surface hydrophobicity. Data are presented as mean ± standard deviation.

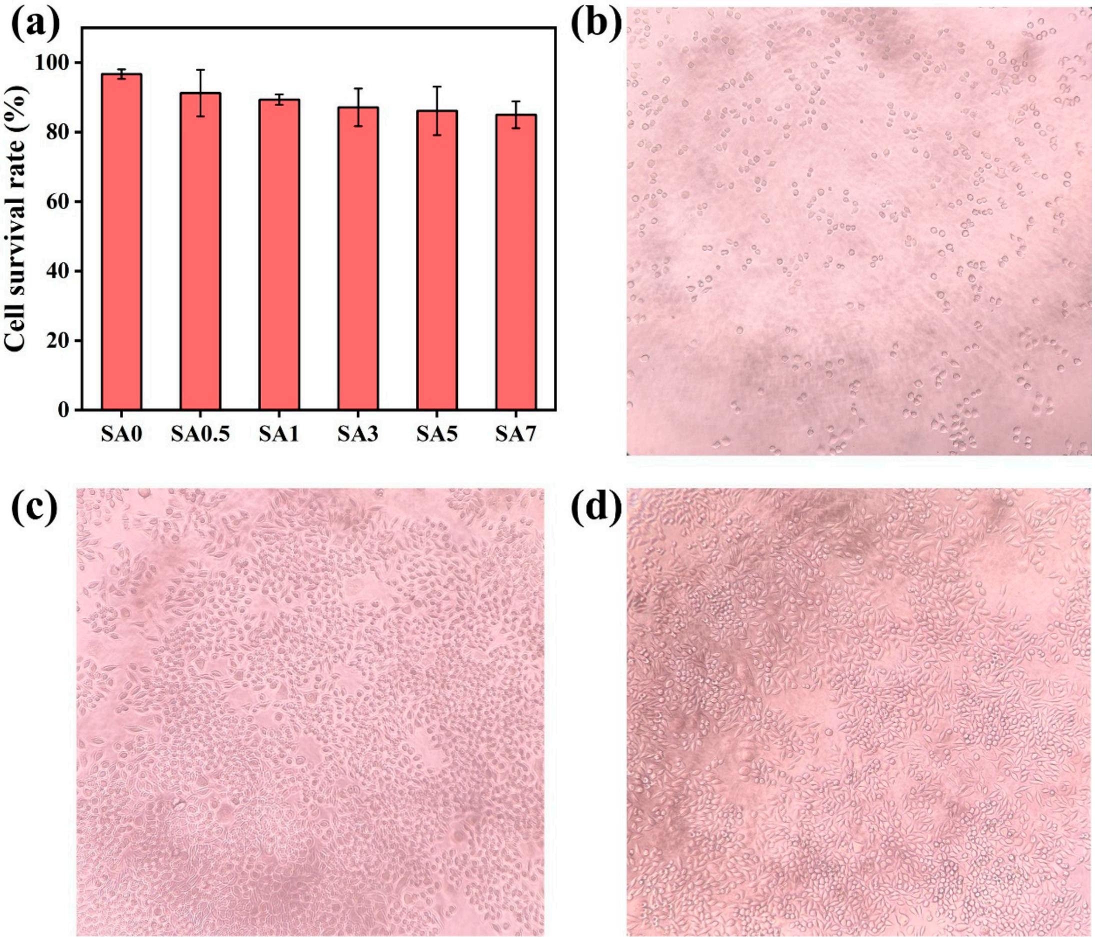

Figure S3. Cytotoxicity assessment of SA coatings using the MTT assay. (a) Cell survival rate (%) of L929 mouse fibroblasts after 48h co-culture with coating solutions (SA0 to SA7). Data are presented as mean ± standard deviation. All formulations showed survival rates above the 80% cytotoxicity threshold. (b) Microscopic image (100x magnification) of L929 cells at the start of the experiment (0h). (c) Microscopic image (100x) of the control group (cells in medium only) after 48h, showing a healthy, confluent cell monolayer. (d) Microscopic image (100x) of L929 cells co-cultured with the SA5 coating solution for 48h, demonstrating high cell density and normal morphology, comparable to the control.
